# Retinoic-Acid-Related Orphan Receptor Alpha Is Involved in the Regulation of the Cytoskeleton of Hair Follicle Stem Cells

**DOI:** 10.3390/biom15060863

**Published:** 2025-06-13

**Authors:** Yu Zhang, Xuefei Zhao, Shuqi Li, Suying Bai, Wei Zhang

**Affiliations:** 1 College of Wildlife and Protected Area, Northeast Forestry University, Harbin 150040, China; zhangyu2817@nefu.edu.cn (Y.Z.); zhaoxuefei@nefu.edu.cn (X.Z.); graceli@nefu.edu.cn (S.L.); 2 National Forestry and Grassland Administration Research Center of Engineering Technology for Wildlife Conservation and Utilization, Harbin 150040, China; 3 Detecting Center of Wildlife, State Forestry and Grassland Administration, Harbin 150040, China

**Keywords:** *Rorα*, hair follicle stem cell, cytoskeleton

## Abstract

The development and replacement of hair play a significant role in the life history of animals. In recent years, retinoic-acid-related orphan receptor alpha (*Rorα*) has been found to participate in the regulation of hair follicle development, yet the underlying mechanisms remain incompletely understood. This study aims to analyze the regulatory role of *Rorα* on the cytoskeleton of hair follicle stem cells (HFSCs). We treated HFSCs with a RORA agonist and subsequently analyzed differential gene expression using qPCR, Western blotting, and immunofluorescence, finding that agonist-induced activation of RORA suppressed the expression levels of cytoskeleton-related genes. Additionally, F-actin staining with phalloidin, followed by migration assays and wound healing tests for cell migration detection, revealed that this process affected the cytoskeletal state of HFSCs and inhibited their migration and adhesion capabilities. We further conducted interaction analyses using CUT&RUN combined with ddPCR and EMSA, demonstrating that RORA can bind to the promoter regions of the *Actg1* gene and regulate their transcription. This study contributes to a comprehensive understanding of the regulatory processes involved in hair follicle development and may provide broader insights into the treatment of diseases such as alopecia.

## 1. Introduction

Hair is a unique keratinized organ of mammals. Current views suggest that hair plays a crucial role in mechanical protection, thermal insulation, secondary sexual characteristics, sensation, and other aspects. Therefore, normal hair development is an important guarantee for animals to survive in harsh environments [1,2,3]. Hair is composed of numerous relatively independent hair follicle structures. A standard hair follicle comprises various structures, including the hair bulb, hair shaft, arrector pili muscle, sebaceous gland, inner root sheath, and outer root sheath [4,5,6]. The hair follicles of animals exhibit a cyclical developmental process, which is generally categorized into three distinct stages based on morphological features: the anagen phase, the catagen phase, and the telogen phase [7,8]. In the life history of many animals, the maintenance and renewal of hair occupy an important position, and the maintenance and renewal of hair follicles are influenced by both endogenous and exogenous factors. The endogenous factors affecting hair follicle development include its own developmental cycle and other endogenous factors such as hormone levels [9,10,11,12,13]. Exogenous factors, on the other hand, mostly refer to environmental factors such as temperature and photoperiod [14,15]. A fully developed hair follicle comprises as many as several dozen distinct cell types. It is widely accepted that HFSCs are pivotal to the cyclical regeneration of hair follicles. The periodic activation of these stem cells serves as the cellular foundation for this regenerative process. HFSCs primarily reside in the bulge region, which is located at the intersection of the arrector pili muscle and the outer root sheath [16,17]. They possess the characteristics of adult stem cells, capable of participating in both hair follicle development and migrating upwards to participate in the repair of skin injuries. Therefore, they serve as an excellent entry point for studying the process of hair follicle development and the treatment of diseases such as alopecia [18,19].

Melatonin serves as an important bridging molecule in organisms’ response to changes in photoperiod [20]. Additionally, melatonin is also a crucial component in the response of organisms to both circadian and seasonal rhythms. Numerous studies have demonstrated that melatonin has a significant impact on the developmental regulation of animal hair and hair follicles [21,22,23,24]. Meanwhile, studies on humans have also shown that melatonin has significant therapeutic effects in the treatment of human hair loss. Research involving patients with androgenic alopecia and diffuse alopecia has demonstrated that the topical administration of 0.1% melatonin over a six-month period can substantially elevate the proportion of hair follicles in the anagen phase, thereby offering promising new therapeutic strategies for addressing hair loss [25]. Another study on androgenic alopecia has demonstrated that topical melatonin treatment exhibits significant improvement in androgenic alopecia, with good safety and tolerability profiles. It can serve as a valuable complement to existing hair loss treatments such as finasteride and minoxidil [26]. Although preliminary discussions have been conducted on the therapeutic value of melatonin in treating diseases such as alopecia, unfortunately, a comprehensive characterization of the underlying detailed molecular mechanisms is still lacking. A significant pathway for melatonin to exert its physiological effects is through signal transduction mediated by its receptors. Therefore, research into melatonin-receptor-mediated physiological effects can help uncover the regulatory relationship between melatonin and hair follicle development.

Retinoic-acid-related orphan receptor alpha (*Rorα*) is a member of the steroid hormone receptor subgroup within the broader nuclear receptor superfamily and is involved in multiple physiological and pathological processes [27]. Moreover, *Rorα* is identified as a nuclear receptor for melatonin, and it plays a pivotal regulatory role in mediating many of melatonin’s biological effects [28]. As the nuclear receptor for melatonin, RORA’s role in hair follicle development is gradually being characterized. Studies have shown that RORA may be involved in the seasonal shedding of wool in sheep [29]. Our previous research has also verified that RORA can affect the apoptosis level of hair follicle stem cells in rats by regulating the expression of the *Bnip3* gene [30]. However, the discussion surrounding the regulatory role of RORA in hair follicle development is still in its preliminary stage. Further research is needed to provide us with a comprehensive understanding of the regulatory process of hair follicle development.

In a narrow sense, the cytoskeleton refers to the protein fiber network system in eukaryotic cells, consisting of microtubules, microfilaments, and intermediate filaments. In a broader sense, the cytoskeleton system is considered as a network system formed by the nuclear skeleton, cytoplasmic skeleton, plasma membrane skeleton, and extracellular matrix [31,32]. The cytoskeleton system has a complex protein composition, with widely recognized protein types including actin, myosin, tropomyosin, and tubulin. The cytoskeleton system plays a crucial role in maintaining cell morphology and the orderliness of internal structures. Additionally, it is involved in a series of important physiological activities such as cell division, intracellular material transport, cell migration, and adhesion [33,34,35,36]. The proper migration of hair follicle stem cells is crucial in the process of hair follicle reconstruction, and existing research has revealed that abnormal migration and depletion of hair follicle stem cells are significant contributors to hair follicle degeneration [37]. Given the role of the cytoskeleton system in regulating a range of cellular physiological effects, including cell migration, this study aims to utilize rat hair follicle stem cells as a cellular model to investigate the impact of the RORA protein on their cytoskeleton, thereby providing a theoretical basis for research related to the regulation of hair follicle development. Additionally, it may offer potential therapeutic targets and theoretical support for the treatment of alopecia and other related issues.

## 2. Materials and Methods

### 2.1. Cellular Model and Drug Treatment

The cellular model employed in this study consisted of rat hair follicle stem cells that had been maintained in long-term storage in the laboratory, and the isolation technique utilized was based on the methodologies outlined in research conducted by Oshima, Rochat, and Shwartz et al. [38,39,40]. The cell isolation procedure is briefly outlined as follows: First, skin tissue was harvested from the snout area of 8-week-old male Sprague–Dawley rats. Under a dissecting microscope, the dermis and other connective tissues were carefully removed to expose the hair follicle structures. Using micro-neural forceps and a 1 mL syringe needle, individual hair follicle tissues were isolated, and the connective tissue sheath of each follicle was longitudinally incised. After excising additional tissues such as the hair bulb, hair shaft, and arrector pili muscle, the bulge region of the hair follicle was extracted for primary culture using the tissue explant method. The HFSCs procedure was performed in Dulbecco’s Modified Eagle Medium/Nutrient Mixture F-12 supplemented with 2% fetal bovine serum (Gibco, San Francisco, CA, USA, A5256701), GlutaMAX™ (Gibco), penicillin–streptomycin, 50 ng/mL of epidermal growth factor, and basic fibroblast growth factor, maintained at 37 °C with 5% CO_2_. The cells were identified based on surface markers of HFSCs reported in the literature (*CD34*, *Krt15*, *CD29*, *CD200*, *CD324*, and *Nestin*), with a purity exceeding 90%. Given that melatonin, an endogenous ligand of RORA, is present under physiological conditions, the control group in this study was treated with a final concentration of 1000 ng/L melatonin to better mimic the physiological state. The experimental group, in addition to receiving 1000 ng/L melatonin, was further treated with a final concentration of 10 μM of RORA agonist SR1078 (MCE, MonmouthJunction, NJ, USA, HY-14422) to amplify RORA’s effects. All cells used in this study were prepared from a single batch and originated from the same cell source, with a drug treatment duration of 24 h.

### 2.2. Immunofluorescence

Cell slides were prepared, and three replicate groups were established. The cells were rinsed thoroughly multiple times with PBS (phosphate-buffered saline) to eliminate any residual medium and cellular debris. After fixing the cells with acetone for 15 min, they were permeabilized using 0.5% Triton X-100 at room temperature for 20 min. Following a 30 min blocking step with goat serum at room temperature, the primary antibody was added, and the slides were incubated overnight at 4 °C. The next day, after removing the primary antibody and rinsing with PBS several times, the secondary antibody was added and incubated at room temperature for 1 h (Proteintech, Rosemont, IL, USA, SA00003-1, SA00003-2, 1:100). After incubation, the cells were rinsed again several times to thoroughly remove the secondary antibody. The slides were then mounted with an anti-fluorescence quenching mounting medium containing DAPI and moved to a fluorescence microscope for observation and result acquisition.

### 2.3. Real-Time qPCR

The cell culture medium was aspirated, and the cells were gently rinsed with PBS. Subsequently, the cells were digested using 0.25% trypsin containing EDTA, which had been pre-warmed to 37 °C, and collected as a cell pellet. RNA extraction was performed using a Thermo Scientific GeneJET RNA Kit (Thermo Fisher, Waltham, MA, USA, K0732), and cDNA synthesis was carried out using a PrimeScript™ RT reagent kit with gDNA Eraser (Takara, Kyoto, Japan, RR047A), strictly adhering to the manufacturer’s protocols. qPCR reactions were conducted using SsoAdvanced™ Universal SYBR^®^ Green (Bio-Rad, Hercules, CA, USA, 1725270), with the reaction mixture and cycling conditions set according to the manufacturer’s guidelines. Detection was performed using a Bio-Rad CFX384 Real-Time PCR Detection System, and the instrument’s default settings were utilized for melting curve analysis. *Ppib* served as the internal reference gene for normalization, and transcriptional-level differences were statistically analyzed using the 2^−ΔΔCt^ method. The experiment included three biological replicates, each with three technical replicates. The primer sequences are detailed in the Supplementary Materials.

### 2.4. Western Blotting

The experiment comprised three replicate groups. Following established protocols, cell pellets were isolated from each group and suspended in RIPA (Radio Immunoprecipitation Assay) lysis buffer supplemented with 1 mmol/L phenylmethanesulfonyl fluoride. The resulting mixture was maintained on ice for 10 min, undergoing intermittent vortexing. After centrifugation, the supernatant was collected to obtain the total cellular protein fraction. For nuclear protein extraction, NE-PER Nuclear and Cytoplasmic Extraction Reagents (Thermo Fisher, 78833) were employed, adhering to the manufacturer’s guidelines. Protein quantification was conducted using a BCA Protein Concentration Assay Kit (Solarbio, Beijing, China, PC0020). Following volume adjustment, 4 × Protein SDS-PAGE Loading Buffer (Takara, Kyoto, Japan, 9173) was incorporated and mixed, with subsequent incubation at 99 °C for 10 min to ensure full protein denaturation. Proteins were resolved using SDS-PAGE gels and transferred onto PVDF membranes. Blocking was performed using 5% BSA at room temperature for 1 h, followed by the addition of the primary antibody and overnight incubation at 4 °C. The following day, the primary antibody was aspirated, and the membranes were extensively washed with TBST buffer. The secondary antibody (Proteintech, Rosemont, IL, USA, SA00001-1, SA00001-2, diluted 1:10,000) was then applied and incubated at room temperature for 1 h. After a final round of TBST washing, the membranes were developed using the ECL (enhanced chemiluminescence) method and imaged on a Bio-Rad ChemiDoc MP Imaging System. Band intensity analysis was performed using the ImageJ software (v1.8.0). Comprehensive details regarding the antibodies are provided in the Supplementary Materials.

### 2.5. Migration Experiment

The experiment established three replicate groups, each inoculated with an equal number of cells in a migration chamber featuring 8.0 μm pores. The experimental group received a final concentration of 10 μM SR1078. After a 24 h incubation, the culture medium was aspirated. Cells on the upper surface of the filter membrane were carefully removed using a cotton swab, and the membrane was subsequently detached using a syringe needle. The cells on the lower surface of the membrane were fixed with 4% paraformaldehyde for 15 min and then mounted using an anti-fade mounting medium containing DAPI. The results were examined under a fluorescence microscope by focusing on the central region of the filter membrane.

### 2.6. Wound Healing Assay and Adhesion Capacity Detection

Cells were seeded in culture dishes at a high density, and three replicates were set up, allowing the cells to proliferate until the dishes were completely covered. A 200 μL pipette tip was used to create scratches perpendicular to the surface of the dish. The dishes were then washed three times with PBS to remove detached cells. The experimental group received 10 μM SR1078. After 24 h, the scratch areas were compared. The formula for calculating the cell migration rate based on the wound healing assay experiment is as follows: cell migration rate = (0 h scratch area–48 h scratch area)/0 h scratch area × 100%.

For adhesion capacity detection, cells seeded in six-well culture plates were treated according to the experimental groups. Following 5 h of normal culture, cells were fixed with 4% paraformaldehyde for 15 min. DAPI staining was then performed, and the adherent cells were counted using a fluorescence microscope. Three different fields of view for cell counting were selected, and the relative proportion of CG and EG cell numbers was calculated.

### 2.7. Phalloidin Staining

Following drug treatment, the culture medium was aspirated, and the cells were rinsed with PBS buffer. The cells were then fixed with 4% paraformaldehyde at room temperature for 30 min. After removing the fixative, the cells were washed again with PBS buffer and permeabilized with 0.5% Triton X-100 for 5 min. The permeabilization solution was aspirated, and the cells were washed once more. Subsequently, the cells were incubated with FITC-labeled phalloidin (Bipsharp, Hefei, China, BL1188A, 100 nM) in the dark at room temperature for 30 min. Following incubation, the cells were washed with PBS to eliminate any residual phalloidin, mounted with an anti-fade mounting medium containing DAPI, and visualized under a fluorescence microscope.

### 2.8. Cleavage Under Targets and Release Using Nuclease (CUT&RUN)

The CUT&RUN experiment was performed using a CUT&RUN Assay Kit (Cell Signaling Technology, Danvers, MA, USA, #86652, #14209) to enrich DNA fragments bound to the downstream regions of RORA. The procedure adhered strictly to the manufacturer’s instructions. The experimental groups were configured as follows: an experimental group (*n* = 3) treated with RORA-specific antibodies and a control group (*n* = 1) treated with IgG antibodies. The enriched DNA products were subsequently analyzed via ddPCR to assess RORA binding to the promoter regions of target genes.

### 2.9. Droplet Digital PCR (ddPCR)

EvaGreen Digital PCR Supermix (Bio-Rad, #1864034) was utilized for ddPCR detection. Following dilution of the DNA samples, the reaction system was prepared as per the manufacturer’s instructions and transferred to a droplet generation card. Droplet generation oil (70 μL, Bio-Rad, #1864005) was added to the corresponding wells of the card, and droplets were generated using a droplet generator (Bio-Rad, #1864002). After droplet formation, the droplet suspension was transferred to a 96-well plate for PCR amplification, adhering to the reaction program outlined in the kit. Following the reaction, a droplet reader (Bio-Rad, #1864003) and the Bio-Rad QuantaSoft™ Analysis Pro (software version 1.4) software were employed to read and analyze the positive droplets.

### 2.10. Super-Shift Electrophoretic Mobility Shift Assay

A LightShift Chemiluminescent EMSA Kit (Thermo Fisher, 20148) was employed for super-shift EMSA analysis. Probes were incubated with 30 μg of nuclear protein. For competition assays, a 200-fold excess of unlabeled probe (cold probe) and mutant probe were added separately. In the super-shift experiment, a RORA antibody was also included. Nucleic acid–protein complexes were resolved on a 5% non-denaturing polyacrylamide gel.

### 2.11. Statistical Analysis

Each experiment included at least three biological replicates, and Student’s *t*-test was used to assess statistical significance. All statistical analyses were performed using the GraphPad Prism 9.5.1 software, and the results were expressed as mean ± standard deviation. *p* < 0.05 was considered to indicate a statistically significant result.

## 3. Results

### 3.1. RORA Activation Inhibits mRNA Levels of Genes Related to Cytoskeletal Composition and Cell Junctions

**Figure 1:**
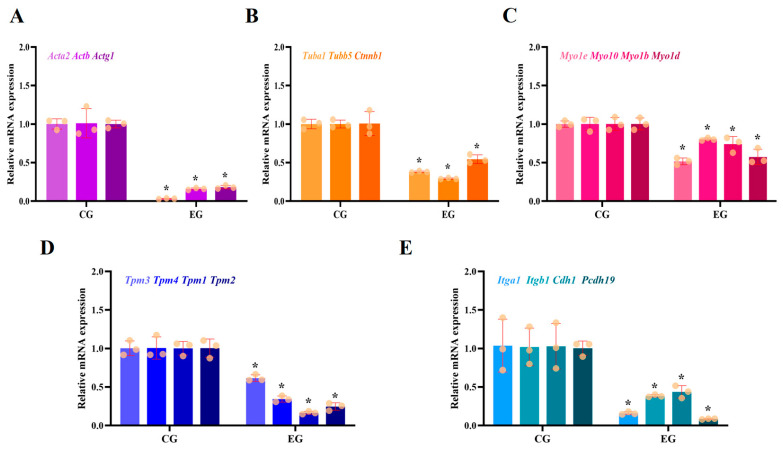
(**A**) Significant differences in transcription levels of *Acta2*, *Actb*, and *Actg1* genes between control group (CG) and experimental group (EG) (*n* = 3, * *p* < 0.05); (**B**) differences in transcription levels of *Tuba1*, *Tubb5*, and *Ctnnb1* between CG and EG groups (*n* = 3, * *p* < 0.05); (**C**) significant differences in the transcription levels of *Myo1e*, *Myo10*, *Myo1b*, and *Myo1d* between CG and EG groups (*n* = 3, * *p* < 0.05); (**D**) statistically significant differences in transcription profiles of *Tpm1*, *Tpm2*, *Tpm3*, and *Tpm4* between CG and EG groups (*n* = 3, * *p* < 0.05); (**E**) differences in transcription levels of *Itga1*, *Itgb1*, *Cdh1*, and *Pcdh19* between CG and EG groups (*n* = 3, * *p* < 0.05).

First, we examined the transcriptional differences in genes involved in cytoskeletal composition. The qPCR results revealed that RORA activation induced by SR1078 downregulated the levels of Acta2, Actb, Actg1, Tuba1, Tubb5, and Ctnnb1 (Figure 1A,B). Specifically, the transcription level of Actg1 significantly reduced by approximately 80% compared to the control group, while Actb transcription declined by about 85% (*p* < 0.05). The transcription levels of Tuba1 and Tubb5 significantly decreased by roughly 60% and 70%, respectively, compared to the control (*p* < 0.05). The mRNA level of Ctnnb1 significantly dropped by about 50%, and notably, the transcription level of Acta2 decreased by 97% compared to the control (*p* < 0.05). These results suggest that RORA activation induced by SR1078 can significantly inhibit the transcription levels of genes involved in cytoskeletal composition, potentially affecting cytoskeletal formation and dynamic regulation.

Next, we investigated the transcriptional levels of some myosin and tropomyosin genes. We found that the mRNA level of Myo1e decreased by about 50% compared to the control, while Myo10 transcription declined by approximately 20% (*p* < 0.05, Figure 1C). The mRNA levels of the Myo1b and Myo1d genes decreased by 25% and 40%, respectively, compared to the control (*p* < 0.05, Figure 1C). Significant decreases were also observed in the transcription levels of some tropomyosin genes, with Tpm3 and Tpm4 mRNA levels dropping by approximately 40% and 60%, respectively, compared to the control (*p* < 0.05, Figure 1D). The mRNA transcription levels of Tpm1 and Tpm2 decreased more markedly, with the experimental group showing only about 15% and 25% of the control levels, respectively (*p* < 0.05, Figure 1D).

Finally, we examined the mRNA levels of the integrin family genes Itga1 and Itgb1, the cadherin family gene Cdh1, and the protocadherin subfamily gene Pcdh19, which are related to cell adhesion (Figure 1E). Similar to the other genes, the transcription levels of these genes in the experimental group also showed significant decreases (*p* < 0.05). The transcription levels of Itga1 and Itgb1 in the experimental group were only about 15% and 40% of the control levels, respectively, while the transcription levels of Cdh1 and Pcdh19 were only about 40% and 10% of the control levels, respectively.

By comparing the transcriptional-level differences of cytoskeleton-related genes before and after the experimental treatments, we discovered that RORA activation exerts extremely significant inhibitory effects on these genes. Notably, genes like Actb and Tubulin, which are involved in cytoskeleton formation, have conventionally been used as housekeeping genes in previous studies related to RORA and melatonin. However, our findings suggest that such selections require more prudent consideration.

### 3.2. RORA Activation Inhibits the Protein Expression of Some Genes Related to Cytoskeletal Composition and Cell Adhesion

**Figure 2:**
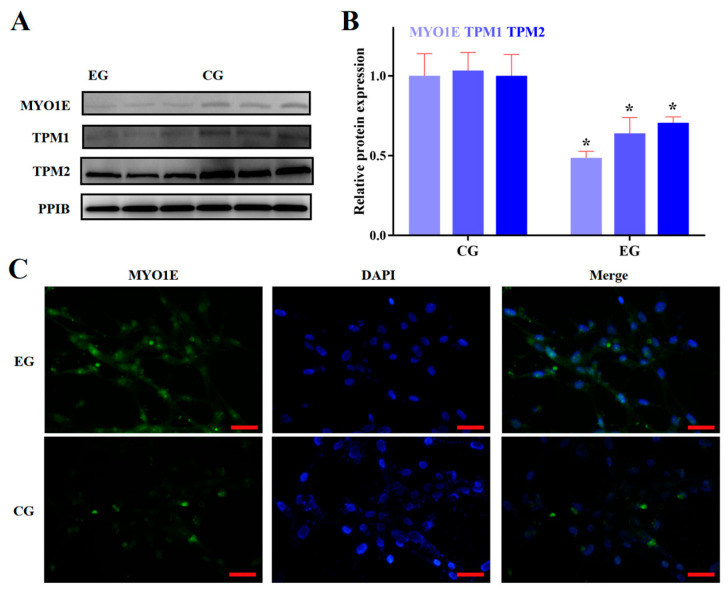
(**A**) Western blot results for MYO1E, TPM1, and TPM2 between control group (CG) and experimental group (EG). (**B**) Relative expression levels of MYO1E, TPM1, and TPM2 proteins between CG and EG groups (*n* = 3, * *p* < 0.05). (**C**) Result of an immunofluorescence assay targeting MYO1E protein (scale bar, 25 μm). Original western blots can be found at Supplementary Materials.

**Figure 3:**
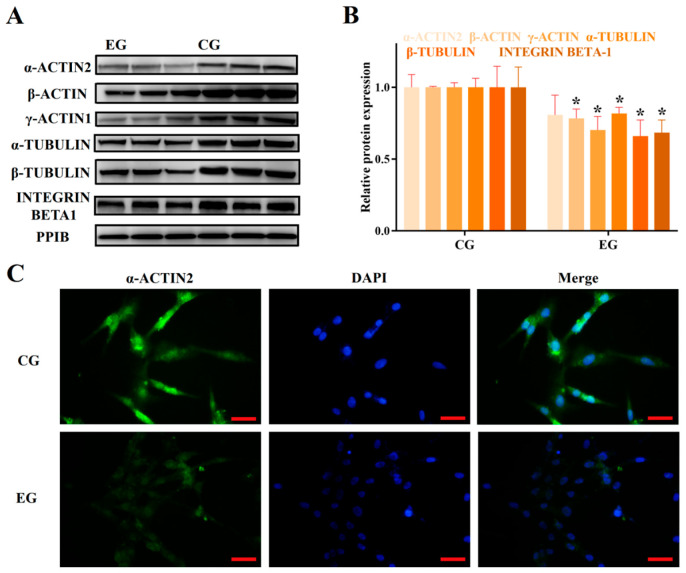

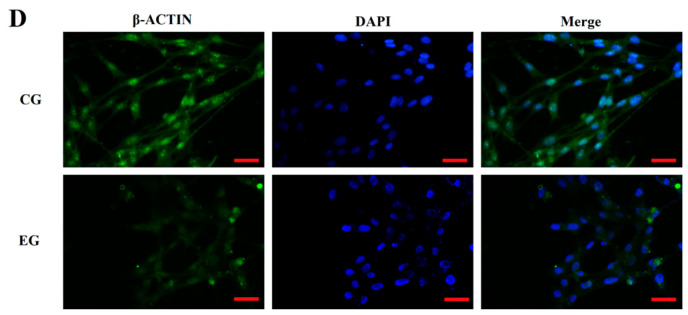
(**A**) Western blot results for α-ACTIN2, β-ACTIN, γ-ACTIN1, α-TUBULIN, β-TUBULIN, and INTEGRIN BETA1 between control group (CG) and experimental group (EG). The same reference gene is shared with Figure 2A. (**B**) Relative expression levels of proteins between CG and EG groups (*n* = 3, * *p* < 0.05). (**C**) Result of an immunofluorescence assay targeting α-ACTIN2 protein (scale bar, 25 μm). (**D**) Result of an immunofluorescence assay targeting β-ACTIN protein (scale bar, 25 μm). Original western blots can be found at Supplementary Materials.

**Figure 4:**
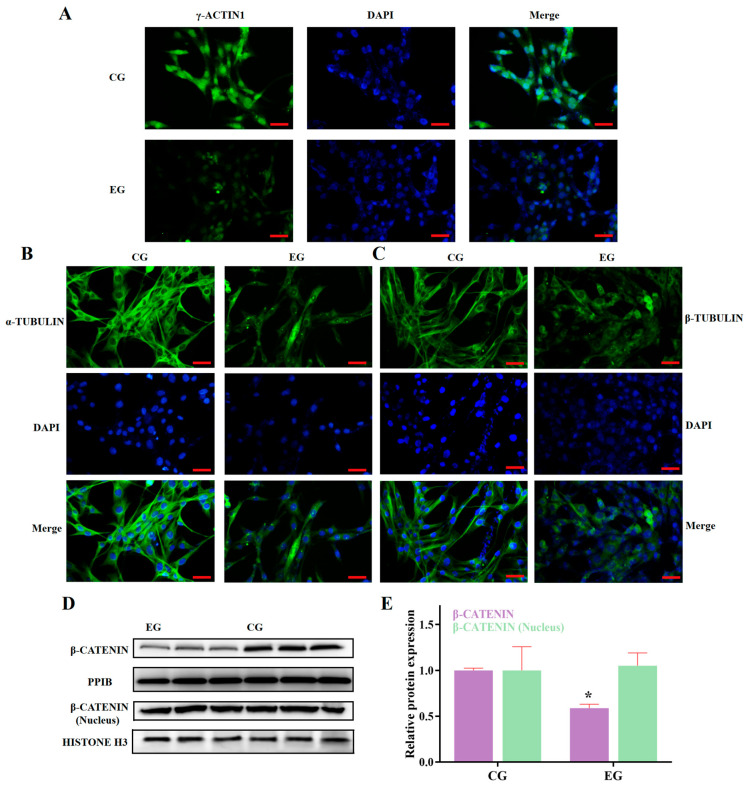
(**A**) Result of an immunofluorescence assay targeting γ-ACTIN1 protein between control group (CG) and experimental group (EG) (scale bar, 25 μm). (**B**) Result of an immunofluorescence assay targeting α-TUBULIN protein (scale bar, 25 μm). (**C**) Result of an immunofluorescence assay targeting β-TUBULIN protein (scale bar, 25 μm). (**D**) Western blot results for β-CATENIN. (**E**) Relative expression levels of β-CATENIN between CG and EG groups (*n* = 3, * *p* < 0.05). Original western blots can be found at Supplementary Materials.

Based on the aforementioned results, we identified the transcriptional inhibitory effect of RORA activation induced by SR1078 on a series of genes. Furthermore, we used WB analysis to detect the protein expression levels of some of these genes (Figure 2A). The experimental results indicated that the protein expression levels of these genes were also suppressed by RORA activation, albeit to a lesser extent than the decrease in mRNA levels. This discrepancy may be attributed to the delayed regulation at the protein level. Compared to the control group, the protein expression of MYO1E in the experimental group significantly decreased by 50%, while the expression of TPM1 and TPM2 proteins decreased by only 40% and 30%, respectively (*p* < 0.05, Figure 2B,C). Among the actin proteins, the protein expression of α-ACTIN2 and β-ACTIN decreased by approximately 20% compared to the control group, while the protein expression level of γ-ACTIN1 decreased by about 30% (*p* < 0.05, Figure 3A,B). For tubulin proteins, the protein expression level of α-TUBULIN was about 80% of the control group, and the β-TUBULIN protein level was about 65% of the control (*p* < 0.05, Figure 3A,B). Additionally, the protein level of INTEGRIN BETA1, which is involved in cell adhesion, significantly decreased by about 30% (*p* < 0.05, Figure 3A,B). We also used immunofluorescence techniques to detect the protein expression of α-ACTIN2, β-ACTIN, γ-ACTIN1, α-TUBULIN, and β-TUBULIN in hair follicle stem cells of both the experimental and control groups (Figure 3C,D, Figure 4A,B). The results were consistent with the WB analysis, providing mutual verification. Notably, β-CATENIN protein is worth mentioning among these proteins, as it not only participates in cytoskeletal composition but also serves as a key component in the classical Wnt signaling pathway. Upon activation, β-CATENIN leaves the cytoskeleton and translocates to the nucleus to regulate the transcription levels of downstream genes. In particular, the state of hair follicle stem cells is highly correlated with the Wnt/β-CATENIN signaling pathway. Therefore, in addition to detecting the level of β-CATENIN in total cellular protein, we also isolated nuclear proteins to determine whether RORA activation might affect the state of hair follicle stem cells by regulating the nuclear translocation of β-CATENIN. The final results showed that, compared to the control group, the level of β-CATENIN in total protein decreased significantly in the experimental group, but there was no significant difference in the nuclear β-CATENIN level (*p* > 0.05, Figure 4D,E).

### 3.3. Activation of RORA Inhibits Cytoskeleton Formation, Migration, and Adhesion of HFSCs 

**Figure 5:**
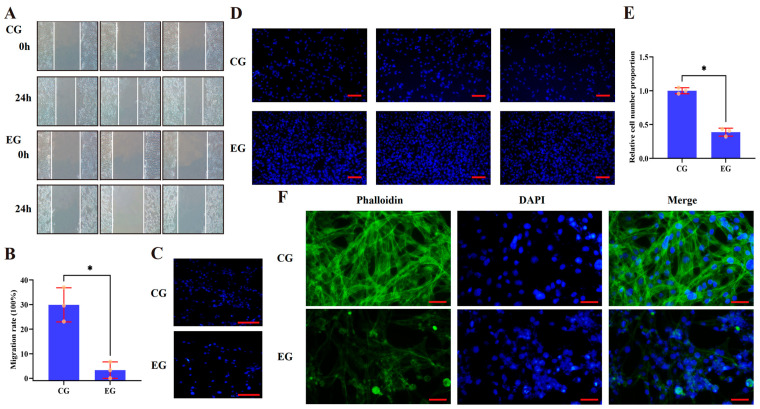
(**A**) The wound healing assay results for the control group (CG) and experimental group (EG) (*n* = 3). (**B**) The statistical results of cell migration rate based on the wound healing assay experiment (*n* = 3, * *p* < 0.05). (**C**) The analysis results of cell migration ability based on transwell assay; scale bar, 50 µm. (**D**) The adhesion assay staining results for the CG and EG groups; scale bar, 50 µm. (**E**) The statistical results of relative cell number of CG and EG in cell adhesion assay (*n* = 3, * *p* < 0.05). (**F**) The phalloidin staining results for the CG and EG groups; scale bar, 25 µm.

After confirming that RORA activation has a significant inhibitory effect on the mRNA and protein expression levels of a series of genes, we conducted migration and wound healing assays to investigate its impact on the migratory ability of HFSCs. The wound healing assay results showed that the wound width in the experimental group was greater than that in the control group (Figure 5A,B). Meanwhile, the migration assay results indicated that RORA activation induced by an agonist significantly reduced the number of cells that migrated through the membrane (Figure 5C). These findings suggest that RORA inhibits cell migration. In the cell–matrix adhesion assay, we found that RORA activation resulted in a decrease in cell adhesion ability, manifested as a reduction in the number of adherent cells (Figure 5D,E). Additionally, the phalloidin staining results revealed that the signal intensity in the experimental group was lower than that in the control group, indicating a decreased number of stable filamentous actin in the experimental group. More interestingly, the staining results of the control group showed clear and distinct structures of stable filamentous actin, whereas many cells in the experimental group lost their typical filamentous actin structure and their adherent, spread morphology, appearing morphologically rounded (Figure 5F).

### 3.4. RORA Can Bind to the Promoter Regions of the Myo1e and Actg1 Genes to Regulate Their Transcription

**Figure 6:**
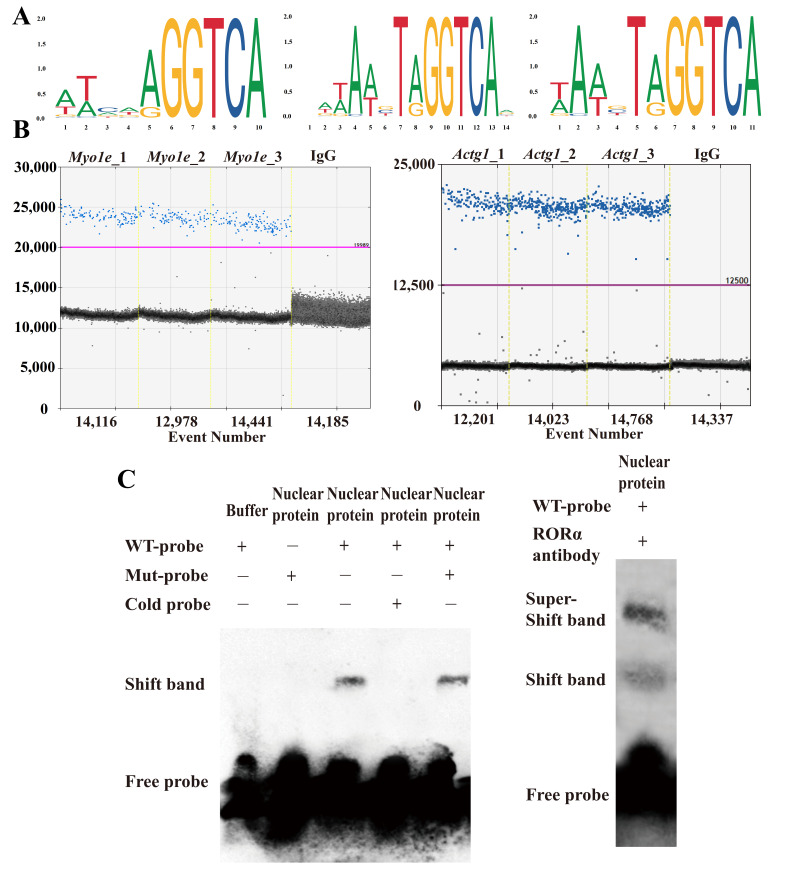
(**A**) Motif structure of RORA protein target genes obtained from the JASPAR database. (**B**) Verification of the presence of promoter fragments of the *Myo1e* and *Actg1* genes in CUT&RUN-enriched DNA samples through tandem ddPCR, with blue dots representing positive droplets and gray dots representing negative droplets. (**C**) Verification of the binding relationship between RORA and the promoter region of the *Actg1* gene using super-shift EMSA.

To further elucidate the interaction between RORA and cytoskeletal components as well as associated genes, we initially enriched DNA fragments corresponding to RORA downstream target binding sites using CUT&RUN technology. We retrieved the motif structures of RORA protein binding to downstream target genes from the Jaspar database and identified a consensus sequence, GGTCA, across these motifs (Figure 6A). This sequence was localized within the promoter regions of the target genes, and specific amplification primers were designed accordingly. Subsequently, we utilized ddPCR to analyze the DNA samples enriched for RORA downstream target binding sites via CUT&RUN. The experimental findings revealed that fragments from the promoter regions of the *Myo1e* and *Actg1* genes were present in the CUT&RUN enrichment products, whereas the IgG control group yielded negative results, suggesting that *Myo1e* and *Actg1* may serve as downstream target genes of RORA (Figure 6B). To further confirm RORA binding to the promoter region of the *Actg1* gene, we employed EMSA technology (Figure 6C). The EMSA results demonstrated that incubation of wild-type probes with nuclear proteins generated shifted bands, which were competitively inhibited by excess unlabeled (cold) probes but not by excess mutant probes. Notably, the inclusion of RORA-specific antibodies induced the formation of super-shifted bands. These findings collectively indicate that RORA binds to the promoter region of the *Actg1* gene.

## 4. Discussion

The development and replacement of hair are pivotal processes in the life history of animals, providing essential protection against harsh environmental conditions. Research into hair development not only enhances our understanding of adaptive evolution but also offers new avenues for treating conditions such as alopecia. Hair follicle stem cells, due to their critical role in hair follicle development and regeneration, hold significant promise as valuable cellular models for investigating the regulatory mechanisms underlying hair follicle growth. *Rorα* is a nuclear receptor widely expressed across various mammalian tissues. However, research into its natural ligands and the physiological effects they mediate has only recently begun to elucidate its full potential [41,42,43,44]. Currently, research on *Rorα* has predominantly centered on its roles in cancer and the nervous system. However, studies investigating its involvement in hair follicle development are increasingly emerging [29,45,46,47,48,49].

In our previous work, we employed CUT&Tag to identify downstream target genes of RORA. Subsequently, we conducted a comprehensive analysis by integrating CUT&Tag and RNA-Seq data to systematically screen for genes that exhibited differential expression after treatment and simultaneously displayed RORA enrichment signals in their promoter regions [50,51]. From these genes, we selected the target genes for this study and focused on investigating the impact of RORA on the cytoskeleton of hair follicle stem cells. By examining the mRNA levels and protein expression of genes related to cytoskeleton composition, we first verified that RORA has a significant inhibitory effect on the transcription and expression of a series of genes. The primary physiological function of the cytoskeleton is to support cell shape. Interestingly, after activating RORA with SR1078, we observed significant changes in cell morphology, with a large number of cells adopting a round shape. This suggests that the inhibition of the expression of a series of cytoskeleton-related genes may have altered the supportive role of the cytoskeleton. Related research findings also support our conclusions. Woods et al. found that stimuli such as cholesterol affect the morphology of primary chondrocytes through RORA, upregulating cell proliferation while rounding the cell morphology. Ultimately, they concluded that cholesterol signaling stimulates chondrocyte hypertrophy through *Rorα* and regulates the response of chondrocyte actin dynamics [52].

Additionally, the cytoskeleton has a significant relationship with the migration ability of cells [53,54]. Cells can achieve the process of migration through the treadmilling behavior of the cytoskeleton (excluding intermediate filaments), which involves assembly at one end and disassembly at the other end [55,56,57,58]. In our study, we found that the activation of RORA also has a notable inhibitory effect on the migration ability of cells. This inhibition of migration may also be highly correlated with changes in the cytoskeleton state, and the inhibition of the expression of the aforementioned proteins may be an important cause of this. Similarly, research in the field of cancer provides support for our findings, with studies on glioblastoma, gastric cancer, and endometrial cancer also identifying an inhibitory effect of *Rorα* on cell migration [59,60,61]. We also discussed the impact of RORA on cell adhesion ability, which encompasses both cell-to-cell adhesion and cell-to-matrix adhesion. In this study, we focused specifically on the adhesion effect between HFSCs and the matrix. We examined the levels of several genes involved in cell adhesion and found that RORA also has an inhibitory effect on them. Additionally, given that the cytoskeleton plays a significant role in cell adhesion ability, we believe that the changes in the cytoskeleton state induced by RORA activation may also be a key reason for the decreased cell adhesion ability [54].

After elucidating the effects of agonist-activated RORA on the cytoskeleton, adhesion ability, and migration ability of HFSCs, we were eager to characterize the underlying molecular mechanisms by which RORA exerts its effects. Therefore, we verified the binding relationship between RORA and *Actg1*, as well as *Myo1e*. The results showed that both *Actg1* and *Myo1e* have the consensus sequence motifs for RORA in their promoter regions, and RORA has the ability to recognize these sequences and bind to their promoters, potentially regulating the transcription levels of both genes. Among the series of genes we examined, not all had RORA motifs in their promoter regions; however, their expression levels showed significant changes after RORA activation. This suggests that RORA may recognize motifs that are currently unknown to us or may indirectly affect the expression levels of these genes through other pathways. Based on the findings of this study, we believe that there exists a series of intricate regulatory networks underlying the modulation of HFSCs by RORA. Further elucidation of the relevant molecular mechanisms will contribute to a comprehensive understanding of the regulatory processes involved in hair follicle development. Additionally, it will provide novel targets for the treatment of diseases such as androgenic alopecia and hair follicle degeneration. However, certain limitations remain in the scope of this study. For instance, we only utilized primarily cultured hair follicle stem cells as an experimental model for the in vitro validation of *Rorα*’s physiological functions. This approach provides an incomplete understanding of the comprehensive physiological regulatory effects of *Rorα*. Future research should incorporate in vivo animal studies to further substantiate and strengthen the conclusions drawn from this study.

## 5. Conclusions

The changes in the migration and adhesion capabilities of HFSCs are key factors influencing hair follicle reconstruction, and the cytoskeleton plays a crucial role in determining the adhesion and migration abilities of cells. This study, by analyzing variations in the expression levels of relevant genes, the interactions between RORA and its downstream target genes, as well as alterations in cell state, confirmed that the activation of RORA inhibits the formation of the cytoskeleton in HFSCs while simultaneously reducing their adhesion and migration capabilities. This finding is of significant importance for further elucidating the underlying mechanisms regulating hair follicle development and at the same time offers new perspectives for the treatment of conditions such as hair loss.

## Data Availability

The original contributions presented in this study are included in the article/Supplementary Materials. Further inquiries can be directed to the corresponding author.

## Supplementary Materials

The following supporting information can be downloaded at: https://www.mdpi.com/article/10.3390/biom15060863/s1, Table S1: The primers used in this research; Table S2: The primary antibody used in this research. File S1: Western Blotting Figures.
