# Supplementary material for: Retinoic-Acid-Related Orphan Receptor Alpha Is Involved in the Regulation of the Cytoskeleton of Hair Follicle Stem Cells
Source: Biomolecules. 2025 Jun 13;15(6):863. doi: 10.3390/biom15060863 (PMC12191399; doi:10.3390/biom15060863)
Supplement: Supplementary file 1 [file biomolecules-15-00863-s001.zip › biomolecules-3635770-supplementary tables.pdf]

Supplementary Materials for "Retinoic acid-related orphan receptor alpha is involved in the regulation of the cytoskeleton of hair follicle stem cells"

Yu Zhang *et al.*

*Corresponding author. Email: zwfur@nefu.edu.cn

This file includes:

Tables S1 to S2.

Table S1. The primers used in this research

Primer Name	Sequence 5'-3'
PpibF	GGCACAGGAGGAAAGAGCAT
PpibR	ACCACATCCATGCCTTCCAG
Actg1F	GTACCCTATTGAGCACGGCA
Actg1R	CGCCCAGATGCATACAAGGA
Acta2F	TTCGTGACTACTGCTGAGCG
Acta2R	TAGGTGGTTTCGTGGATGCC
Tuba1bF	GCCAAGCGACAAGACCATTG
Tuba1bR	TCCTTGCCTGTGATGAGCTG
Tubb5F	GCCAGATCTTCAGACCGGAC
Tubb5R	ATGATGCGGTCGGGGTATTC
Itga1F	CGCCAAGATGAACGAGCCTCTG
Itga1R	AATCTGCTCTCCGCCGAGTGT
Pcdh19F	GGAGAGTGACCAGACCGACAGT
Pcdh19R	CCTCATGTGCTCAGGCGACTTG
Ctnnb1F	CTCCTTCCCTGAGTGGCAAG
Ctnnb1R	GTGTCTCAGGGAACATGGCA
TPM1-F	AGGCCAAGCACATTGCTGAAGA
TPM1-R	CAGCCTGAGCCTCCAGTGACTT
TPM2-F	AGCCAAGCACATCGCTGAGGA
TPM2-R	CGACTTGAGAGCCTGGTCCATG
TPM3-F	GCAGAAGAGGCAGACAGGAAGT
TPM3-R	GCAGCACTCAGACACTTCAGGT
TPM4-F	AGGGTGAGCTGGAGAGAGCAGA
TPM4-R	GCTCCTCTTCCAGGTCGCTACT
Myo1b-F	AGCCACCACCATCGCAGCATA
Myo1b-R	ACGCCTAGCCTCCTCCTTCAAG
Myo1d-F	CGGAGCAGGAGAGCCTTGAGTT

Myo1d-R	GCAGCGTCAGCAATAGCGAAGA
Myo10-F	ACACATCTCCAGCCGCAACAAC
Myo10-R	ATCCTCACCGTCTCCAGCATCC
Myo1eF	GCCAAGACAGTCCGCAACAACA
Myo1eR	CGCCCTCGATGAGCTGGTAGAA
Itgb1F	CAATGAGGGTCGTGTTGGGA
Itgb1R	TAAGCCGTTGGACCTATCGC
ActbF	CCACCATGTACCCAGGCATT
ActbR	CGGACTCATCGTACTCCTGC
Actg1 Wt-Probe for EMSA	TGTGAGAGATGGTCACTAGGTGAGA
Actg1 Mut-Probe for EMSA	TGTGAGAGATAGATGCTAGGTGAGA
Myo1e Wt-Probe for EMSA	CTCTGATTGGTCAGGCCTGTG
Myo1e Mut-Probe for EMSA	CTCTGATTCAATGGGCCTGTG

Table S2. The primary antibody used in this research

Protein	Information	Dilution Ratio
Integrin Beta 1	Proteintech Group 12594-1-AP	1:10000
RORα (CUT&RUN)	Abcam ab256799	1:30
RORα (for EMSA)	Santa Cruz sc-518081	The final concentration is 20 μg/ml
PPIB	Proteintech Group 11607-1-AP	1:5000
Alpha Actin2	Proteintech Group 80008-1-RR	1:50000 for WB, 1:500 for IF
Gamma Actin1	Proteintech Group 11227-1-AP	1:3000 for WB, 1:500 for IF
Beta Actin	Proteintech Group 81115-1-RR	1:10000 for WB, 1:1000 for IF
Alpha Tubulin	Proteintech Group 11224-1-AP	1:5000 for WB, 1:300 for IF
Beta Tubulin	Proteintech Group 10094-1-AP	1:1000 for WB, 1:300 for IF
MYO1E	Proteintech Group 17768-1-AP	1:1000 for WB, 1:100 for IF
TPM1	Proteintech Group 28477-1-AP	1:3000
TPM2	Proteintech Group 11038-1-AP	1:2000
Histone-H3	Proteintech Group 17168-1-AP	1:3000
Beta Catenin	Proteintech Group 51067-2-AP	1:10000
