# Supplementary figures and images for: Retinoic-Acid-Related Orphan Receptor Alpha Is Involved in the Regulation of the Cytoskeleton of Hair Follicle Stem Cells
Source: Biomolecules. 2025 Jun 13;15(6):863. doi: 10.3390/biom15060863 (PMC12191399; doi:10.3390/biom15060863)

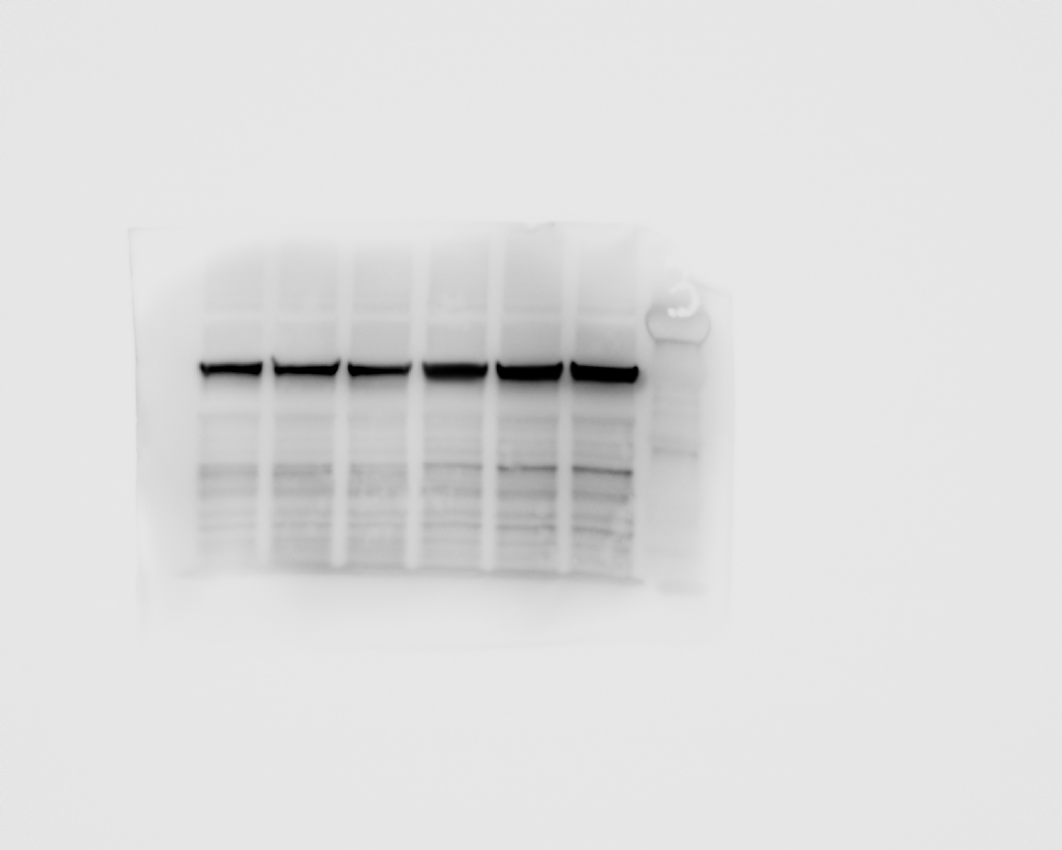

Supplement: Supplementary file 1 [file biomolecules-15-00863-s001.zip › biomolecules-3635770-File S1. original-images/a-tubulin.tif]

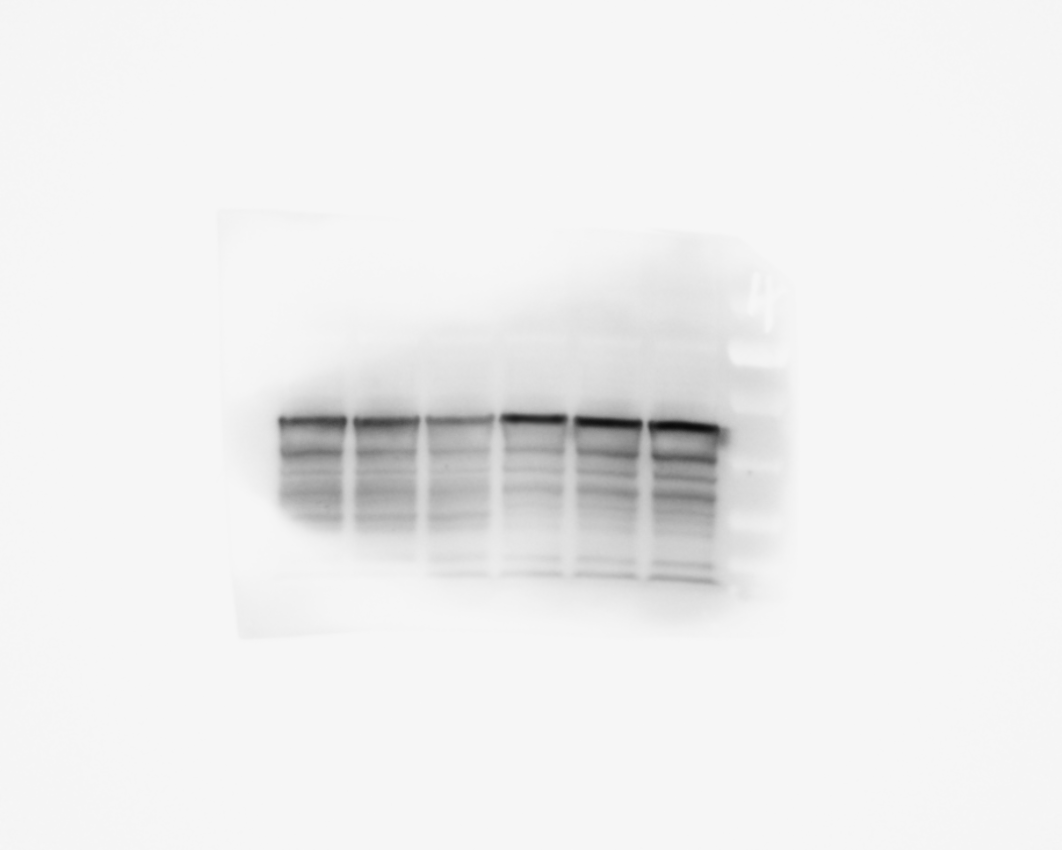

Supplement: Supplementary file 1 [file biomolecules-15-00863-s001.zip › biomolecules-3635770-File S1. original-images/ACTA2.tif]

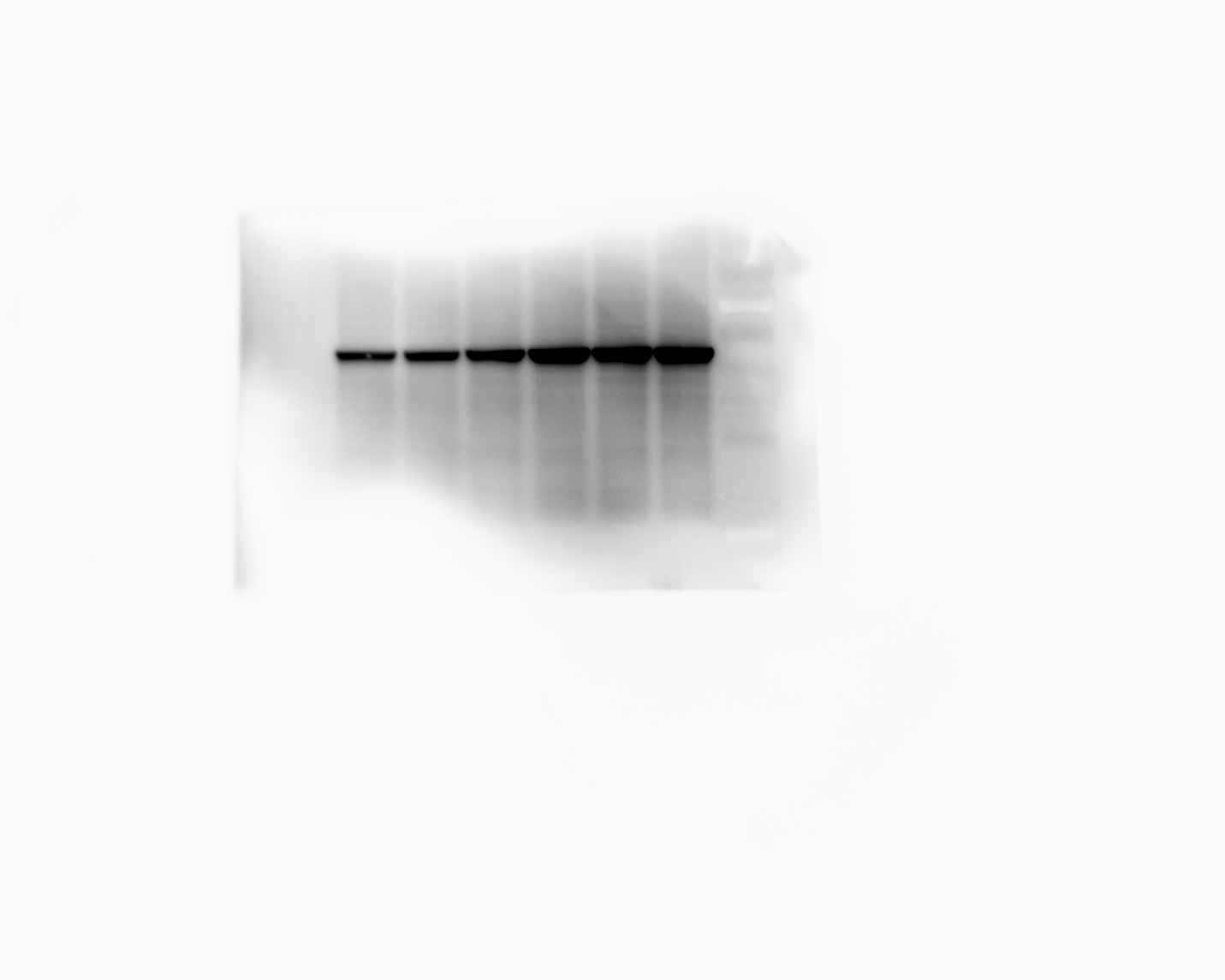

Supplement: Supplementary file 1 [file biomolecules-15-00863-s001.zip › biomolecules-3635770-File S1. original-images/ACTB.tif]

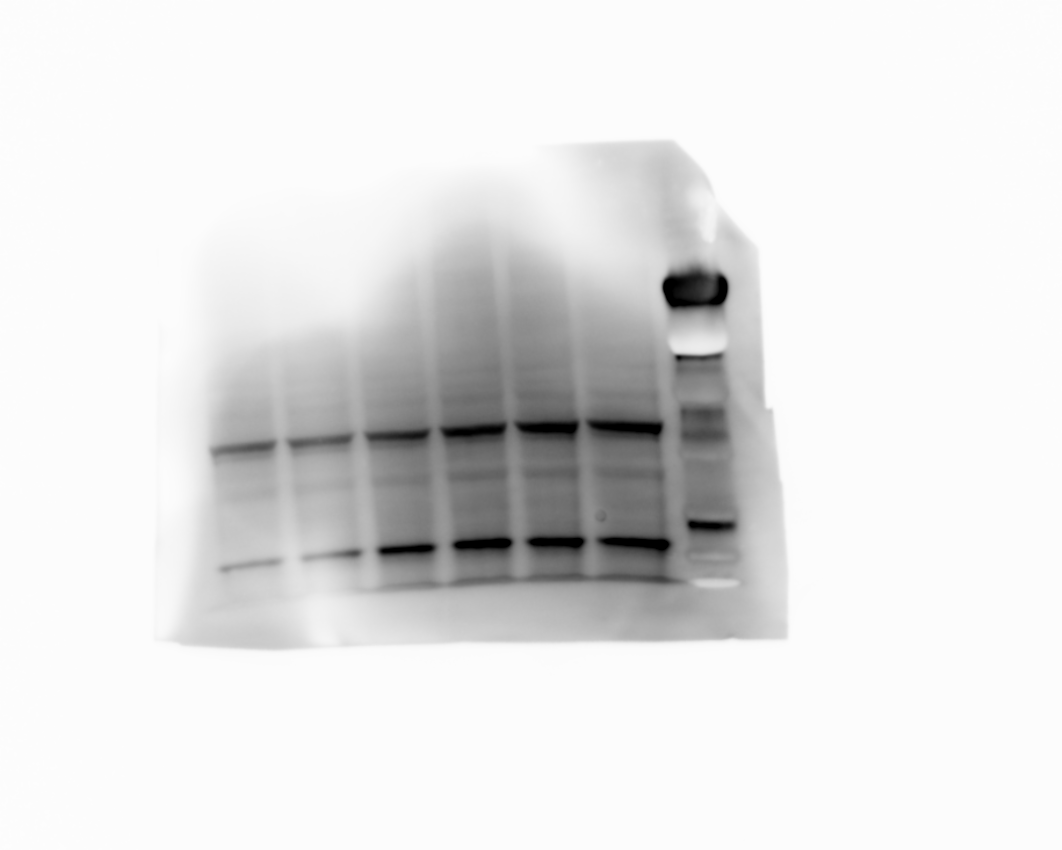

Supplement: Supplementary file 1 [file biomolecules-15-00863-s001.zip › biomolecules-3635770-File S1. original-images/ACTG1.tif]

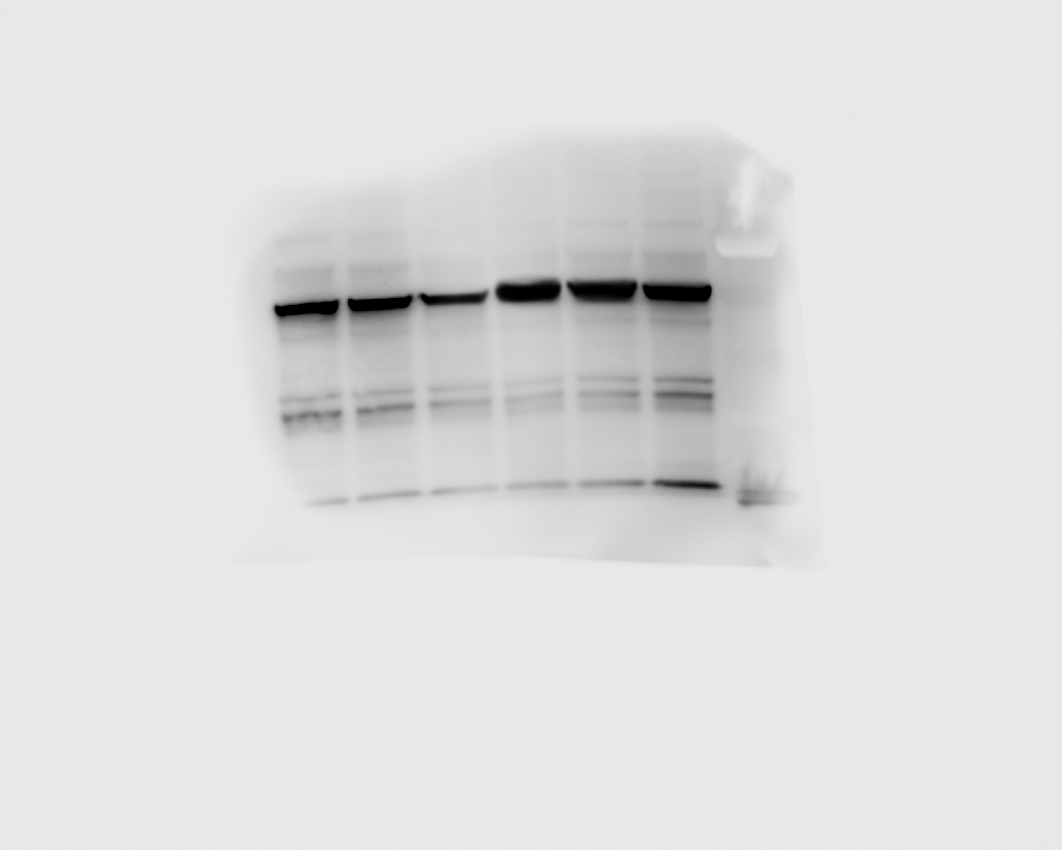

Supplement: Supplementary file 1 [file biomolecules-15-00863-s001.zip › biomolecules-3635770-File S1. original-images/b-tubulin.tif]

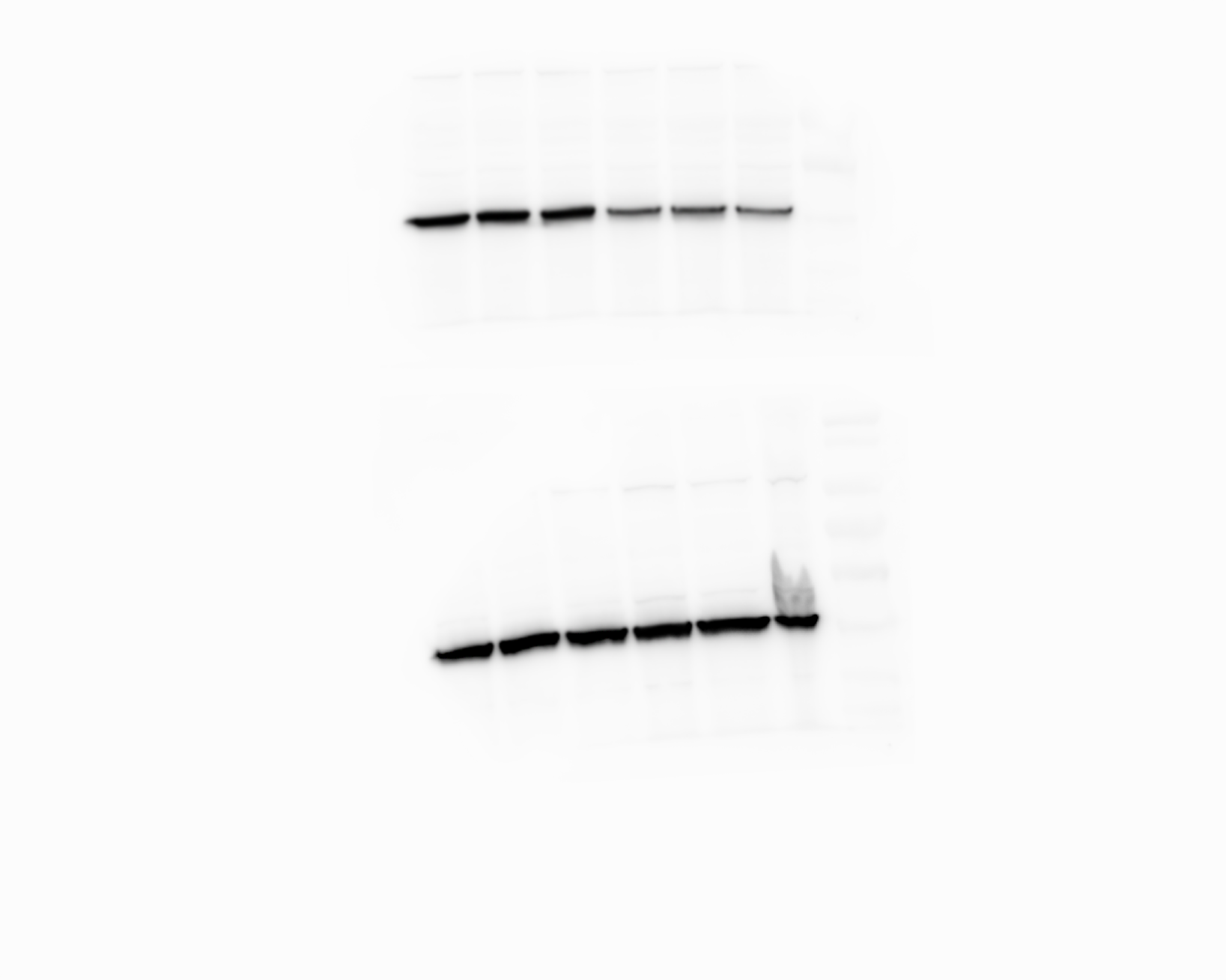

Supplement: Supplementary file 1 [file biomolecules-15-00863-s001.zip › biomolecules-3635770-File S1. original-images/beta Catenin.tif]

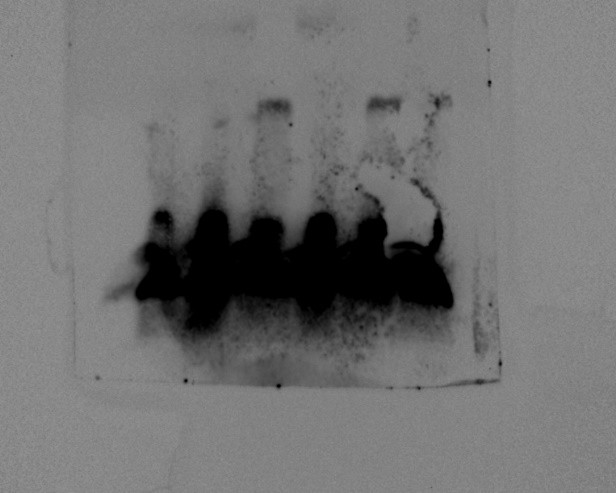

Supplement: Supplementary file 1 [file biomolecules-15-00863-s001.zip › biomolecules-3635770-File S1. original-images/EMSA.jpg]

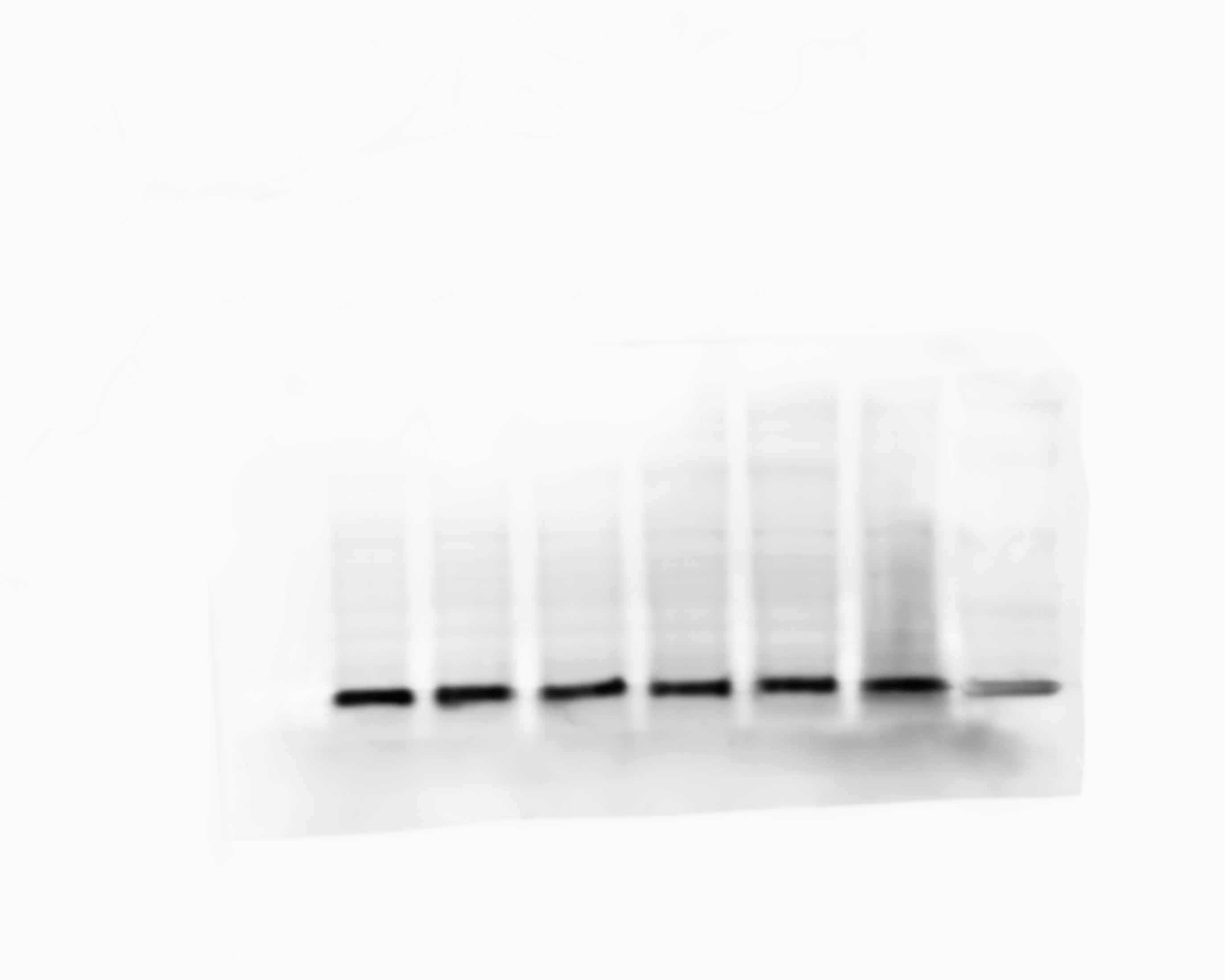

Supplement: Supplementary file 1 [file biomolecules-15-00863-s001.zip › biomolecules-3635770-File S1. original-images/H3 histone .tif]

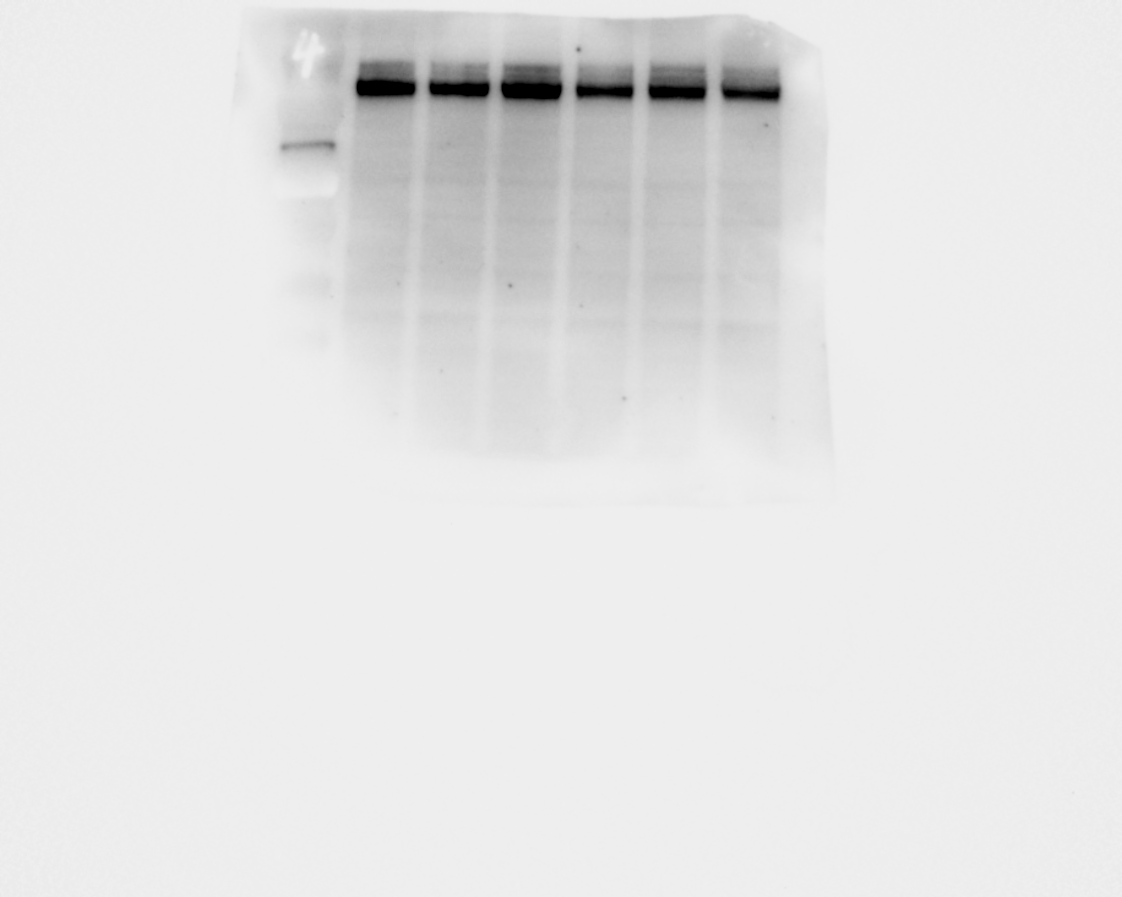

Supplement: Supplementary file 1 [file biomolecules-15-00863-s001.zip › biomolecules-3635770-File S1. original-images/ITGB.tif]

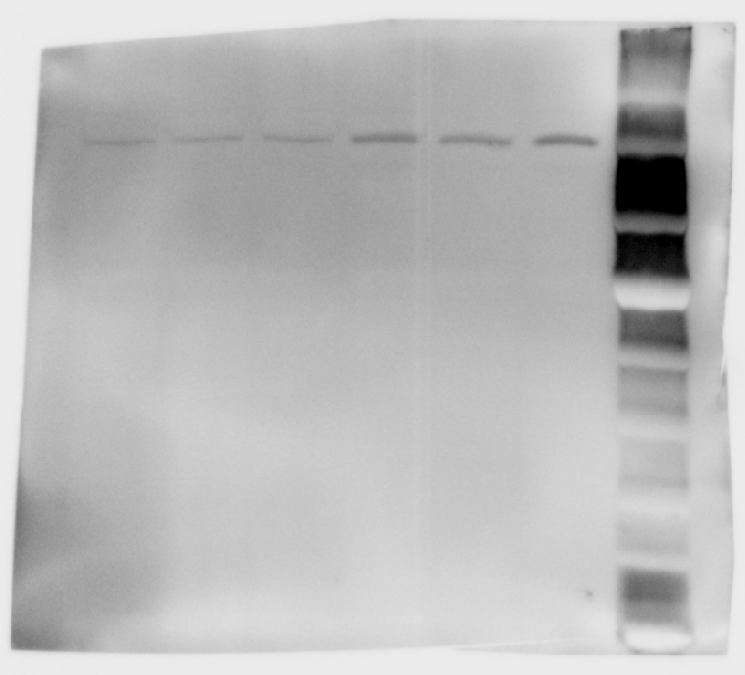

Supplement: Supplementary file 1 [file biomolecules-15-00863-s001.zip › biomolecules-3635770-File S1. original-images/Myo1e.tif]

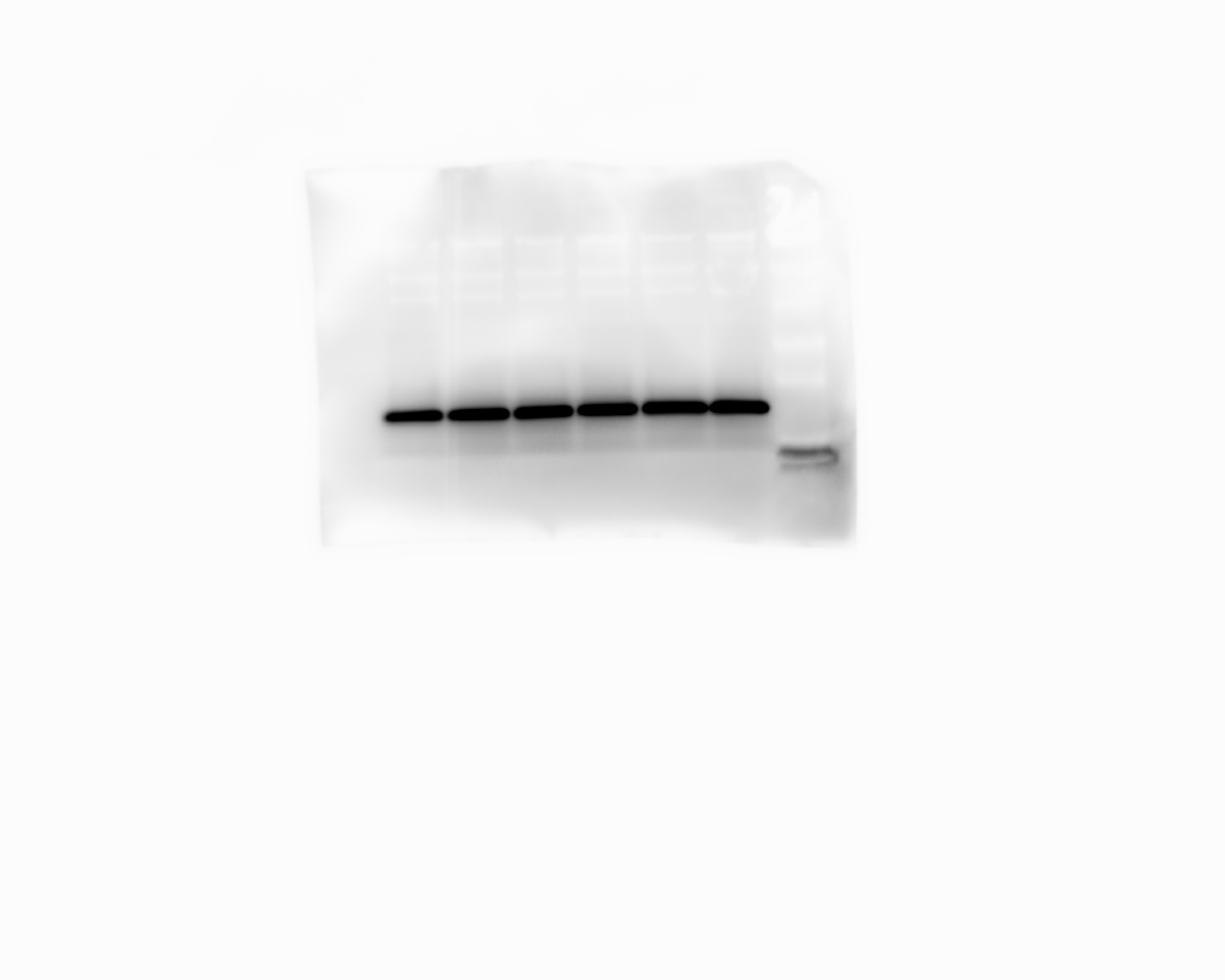

Supplement: Supplementary file 1 [file biomolecules-15-00863-s001.zip › biomolecules-3635770-File S1. original-images/PPIB.tif]

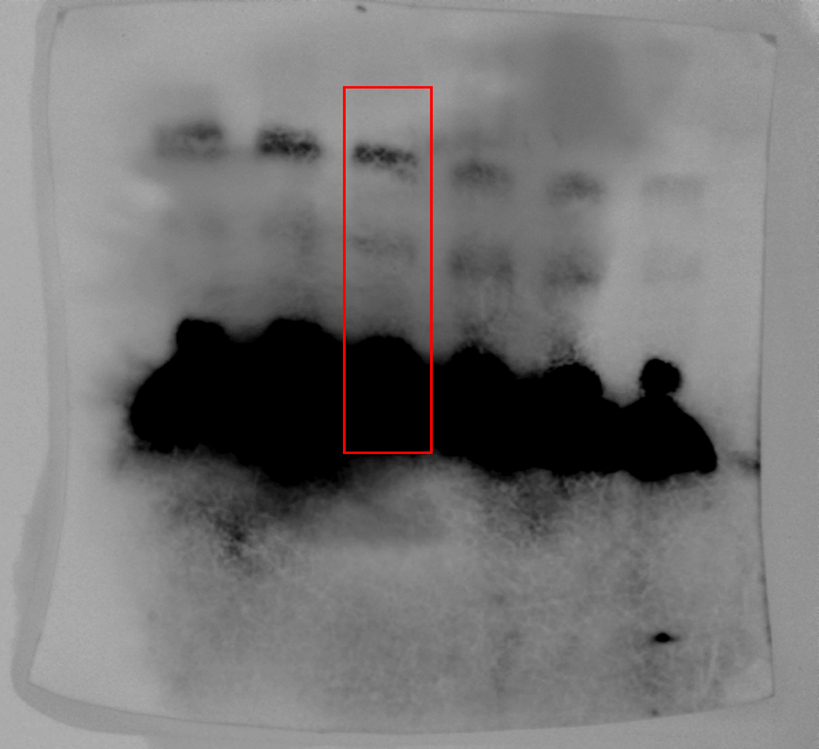

Supplement: Supplementary file 1 [file biomolecules-15-00863-s001.zip › biomolecules-3635770-File S1. original-images/super-shift.png]

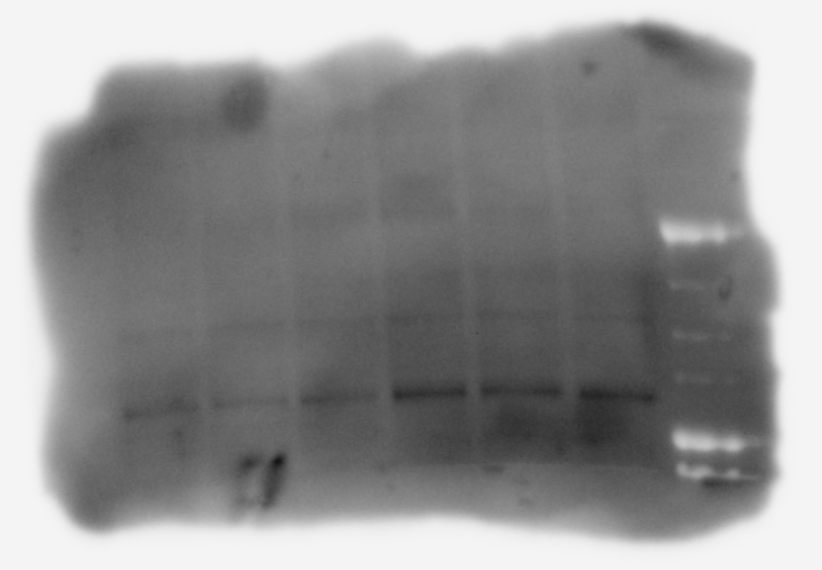

Supplement: Supplementary file 1 [file biomolecules-15-00863-s001.zip › biomolecules-3635770-File S1. original-images/TPM1.tif]

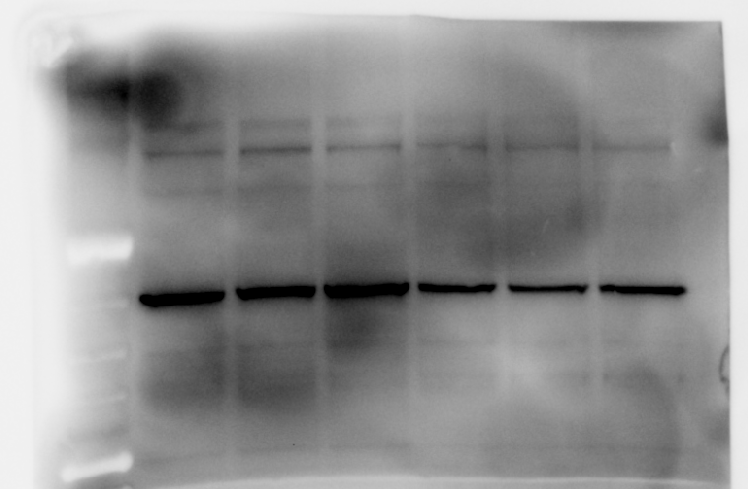

Supplement: Supplementary file 1 [file biomolecules-15-00863-s001.zip › biomolecules-3635770-File S1. original-images/TPM2.tif]
